# Belhassen Tachycardia in a Pediatric Patient: A Simulation for Pediatric Emergency Medicine Fellows

**DOI:** 10.7759/cureus.23521

**Published:** 2022-03-26

**Authors:** Ashley E Keilman, Jason Deen, Julie A Augenstein, Noel Zuckerbraun, Rebekah Burns

**Affiliations:** 1 Pediatrics - Emergency Medicine, University of Washington School of Medicine, Seattle, USA; 2 Pediatrics - Cardiology, University of Washington School of Medicine, Seattle, USA; 3 Pediatrics - Emergency Medicine, Phoenix Children's Hospital, Phoenix, USA; 4 Pediatrics - Emergency Medicine, University of Pittsburgh, Pittsburgh, USA

**Keywords:** belhassen ventricular tachycardia, arrhythmias, simulation-based medical education, pediatric emergency medicine, emergency medicine

## Abstract

Ventricular tachycardia in pediatric emergency department patients is a high-risk, low-frequency event well suited for education through simulation. This technical report describes a simulation-based curriculum for Pediatric Emergency Medicine fellows and senior residents involving the evaluation and management of a 10-year-old female presenting with palpitations who is ultimately diagnosed with Belhassen tachycardia. The curriculum highlights the features that differentiate Belhassen tachycardia (idiopathic left posterior fascicular ventricular tachycardia) from supraventricular or other tachycardias, building upon foundational pediatric resuscitation skills and Pediatric Advanced Life Support (PALS) algorithms for advanced learners.

## Introduction

Due to the relative infrequency of non-sinus tachycardias in Pediatric Emergency Medicine (PEM), exposure to ventricular tachycardias through simulation can help identify and address the knowledge gaps of trainee physicians. Belhassen tachycardia (idiopathic fascicular ventricular tachycardia) is an atypical ventricular tachycardia [1], which requires rapid identification and targeted intervention to prevent progression to hemodynamic decompensation or other comorbid conditions such as tachycardia-induced cardiomyopathy. Standard interventions for supraventricular tachycardia (SVT) such as vagal maneuvers, adenosine, beta-blockers, lidocaine, synchronized cardioversion, and atrial overdrive pacing are ineffective in treating Belhassen tachycardia. Integrating a rapid clinical assessment with diagnostic test results (i.e., ECG) in formulating a management plan and directing resuscitation of tachycardic pediatric patients is a key objective for PEM fellows and senior emergency medicine residents.

Belhassen tachycardia typically presents in older children, adolescents, and young adults and mimics SVT with aberrancy, right bundle branch block, and left anterior hemiblock [2,3]. There have also been case reports in infants and young children [4]. Unlike monomorphic ventricular tachycardia where 90% of cases occur in patients with underlying heart disease, patients who present with Belhassen tachycardia typically have no underlying structural heart disease [5]. A combination of ECG findings including rSR’ V1 morphology, QRS width, positive QRS in aVR and the V6 R/S ratio has been shown to differentiate Belhassen tachycardia from SVT with right bundle branch block and left anterior hemiblock with a sensitivity of 82% and specificity of 78% [6]. An additional key ECG finding is left axis deviation. Belhassen tachycardia can be acutely and chronically managed with verapamil; however, this drug may cause adverse side effects, including hypotension. Particular caution in infants is necessary as their immature myocardium poses an increased risk of verapamil-induced cardiovascular collapse. Intravenous calcium should be immediately administered in all cases to treat verapamil-induced hypotension [7,8].

This technical report was designed for advanced learners, that is, PEM fellows, with strong foundational resuscitation skills and familiarity with Pediatric Advanced Life Support (PALS) algorithms to help them develop a differential diagnosis and management approach for a refractory tachyarrhythmia through the example of Belhassen tachycardia [9]. It would also be appropriate for Pediatric Cardiology fellows and senior Emergency Medicine or Pediatric residents. The case details require learners to recognize the need to deviate from PALS algorithmic management of ventricular tachycardia. While simulation cases that address ventricular tachycardia in pediatric patients are available, there are currently no published resources addressing Belhassen tachycardia [10,11].

## Technical report

Methods

This simulation case was developed by the PEM physicians with expertise in curriculum development and simulation and in consultation with a pediatric cardiologist to complement the existing content of the PEM fellowship simulation curriculum. The scenario was based on an actual patient case. In this participation scenario, participants underwent a rapid patient assessment, interpretation of diagnostic tests, and critical management interventions for Belhassen tachycardia. The simulation was implemented with PEM fellows at three institutions as part of their routine fellow education program. Prerequisite knowledge included an understanding of PALS algorithms [12].

Setting and equipment

This scenario occurred in an emergency department patient room or a simulation lab with a high-technology child manikin. A separate space was used for a debriefing where necessary. The case could be modified to reflect a younger child or older teenager depending upon the availability of the manikin. Medications and equipment typically found in EDs, including medications required to participants for this case were available.

Participants

We implemented this simulation with a total of 18 PEM fellows and two senior Emergency Medicine residents across three training sites. Participants had prior experience with simulation and medical resuscitations. Participants were oriented to the simulator prior to the case if they had not previously worked with that manikin. Each site conducted the simulation once. Due to scheduling constraints, all team roles were filled by physicians.

Personnel

Facilitators were PEM supervising physicians with expertise in simulation development, facilitation, and debriefing methods. The facilitator or simulation specialist provided the voice of the patient. When available a second facilitator acted as the parent. If a single facilitator led the case, they provided the parents’ replies to history questions. A simulation technician familiar with the operation of the child-sized simulator and simulation software managed the simulator.

Pre-briefing

The sessions began with a facilitator-led pre-briefing including a simulation learning contract, orientation to the manikin, and expectation setting for the session including role assignments. The participants were told that a debriefing would be held following the simulation. Participants were given approximately three minutes to huddle to assign team roles.

Case summary

**Figure 1:**
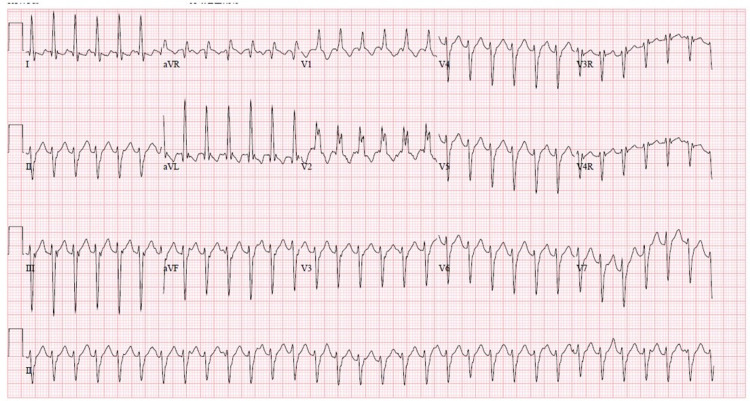
Initial ECG showing wide complex tachycardia with signs of left axis deviation and right bundle branch block consistent with Belhassen tachycardia ECG, electrocardiogram.

**Figure 2:**
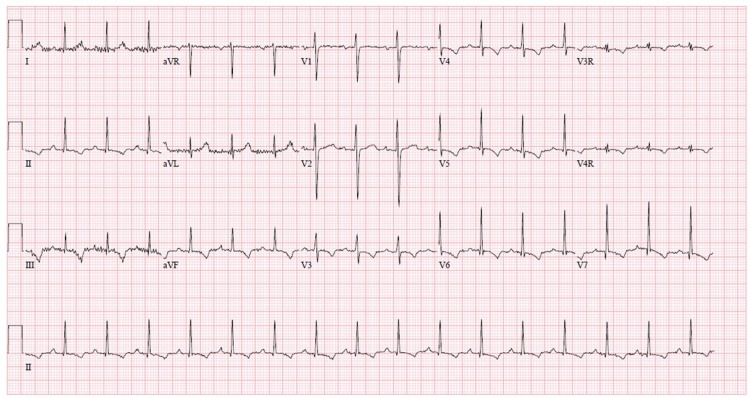
End of scenario ECG showing normal sinus rhythm ECG, electrocardiogram.

**Figure 3:**
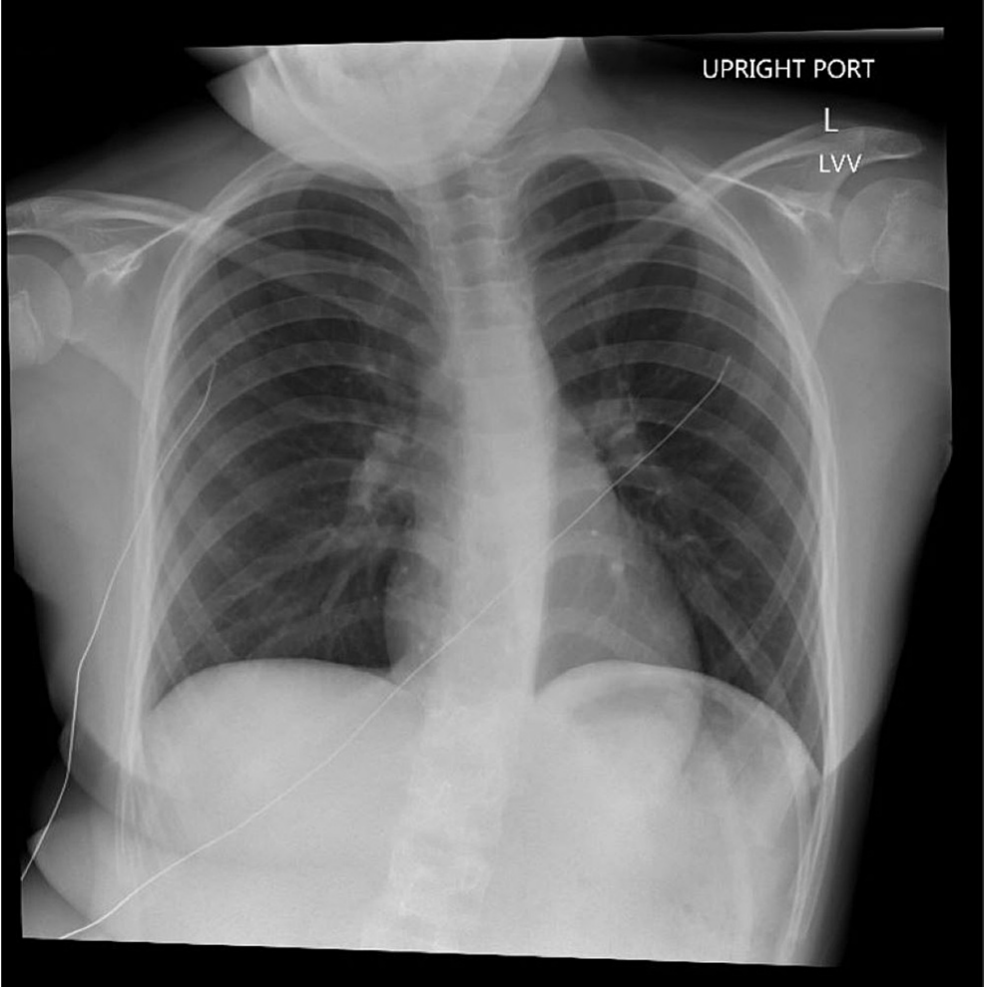
Normal chest X-ray

Facilitators and technicians used a comprehensive, detailed stepwise scenario flowsheet to run the case (Table 1). ECGs (Figures 1 and 2) and a chest X-ray (Figure 3) were available upon request. Throughout the scenario, the simulation facilitator provided additional history and laboratory findings, included in the scenario template, upon request and clinical updates. If using a low-technology simulator, vital signs and physical examination findings may be provided verbally at the learners’ request.

**Table 1:** Stepwise, detailed simulation scenario flowsheet ED, emergency department; HR, heart rate; bpm, beats per minute; BP, blood pressure; RR, respiratory rate; T, temperature; wt, weight; 02 Sat, oxygen saturation; HEENT, head, eyes, ears, nose, throat; PERRLA, pupils equal, round, reactive to light, accommodate; GU, genitourinary; ECG, electrocardiogram; CBC, complete blood count; BNP, B-type natriuretic peptide; EtOH, alcohol; CXR, chest x-ray.

Pre-scenario information	You are working in the pediatric emergency department. A 10-year-old female is brought in by parents for palpitations from her pediatrician’s office
History	
History of presenting illness	A 10-year-old female with a history of eczema is brought in to the emergency department by parents from her pediatrician’s office for evaluation of palpitations. Her symptoms began after playing outside the day before presentation. She was recently ill with cough and nasal congestion, but the symptoms resolved several days ago. The pediatrician noted a very rapid heart rate and referred the patient to the ED. The patient remained awake and alert during the drive to the ED. No interventions were given
Allergies	None
Medications	Topical emollient
Past medical history	Eczema, immunizations up to date
Social history	Lives with parents, in fifth grade
Family history	None
Review of symptoms	A recent mild cough and nasal congestion last week, symptoms now resolved. No fever, difficulty breathing, vomiting, diarrhea, or rashes
Physical examination	
General	Awake, alert, pale, talking
Initial vital signs	HR 209 bpm, BP 95/55, RR 18, T 37.3oC, wt 40 kg, O2 Sat 98%
HEENT	Normocephalic, atraumatic, PERRLA
Neck	Full range of motion
Lungs	Clear to auscultation, normal chest shape, no respiratory distress
Cardiovascular	Regular, tachycardic, HR 205, no murmur, 1+ pulses, cap refill 2-3 s in hands, 3-4 s in feet, central cap refill 2-3 s; no chest wall tenderness to palpation
Abdomen	Soft, non-tender, non-distended, no organomegaly, normal bowel sounds
Neurologic	Awake, alert, moves all extremities, no focal deficits
Skin	Warm, dry, no rashes
GU	Examination deferred
Psychiatric	Cooperative
Stage 1: Initial assessment and diagnostic evaluation	
Expected critical actions: obtain history, physical examination, assess vitals, establish IV access	As above
Request ECG	Facilitator response: ECG pending
If no ECG is requested	If no ECG is obtained, the facilitator or confederate acting as parent states “The pediatrician said she would probably need an ECG”
Stage 2: Identification of Belhassen tachycardia	
Repeat vitals	HR 209 bpm, BP 85/50, RR 18, T 37.3oC, O2 Sat 98%
If labs are requested	If components of electrolytes and complete blood count (CBC) are available on bedside devices and requested by participants, these findings may be shared then. Glucose 114 UA: negative leukocyte esterase, nitrite, glucose, ketones CBC 9.6/13.5/40.1/319 Na 140, K 4.1, Cl 112, Bicarb 14, BUN 12, Cr 0.7, iCal 1.15, Mg 2.0 Phos 3.5 B-type natriuretic peptide (BNP) pending; venous blood gas: 7.28/25/70/17/-5; lactate: 4.5; urine toxicology screen negative; serum EtOH, acetaminophen, salicylate levels: negative
If ECG is requested	Provide initial ECG (Figure 1) shows: right bundle branch block, left axis deviation, QRS 130 ms, positive aVR
IF a CXR is requested	Participants are shown a normal chest X-ray (Figure 3)
Stage 3: Management of Belhassen tachycardia	
If a cardiology consult is requested	Facilitator responds: cardiology will call back in 5 min
If adenosine, beta-blockers, amiodarone, lidocaine procainamide are given	No change in vitals or rhythm
If calcium gluconate or chloride are administered	No change in cardiac tracing
If the patient is cardioverted with 0.5-1 J/kg	The patient briefly returns to sinus tachycardia with pulses but then re-enters rhythm and 80/50. If the patient does not receive pain medications before cardioversion, the patient screams, “Ouch! That really hurts”
Repeat ECG obtained after verapamil	Participants are shown an ECG with rate 96, QRS 110 msec, normal axis (Figure 2)
Repeat vitals after verapamil	HR 149 bpm, BP 100/65, RR 18, T 37.3oC
If participants do not give verapamil or do not arrive at Belhassen tachycardia diagnosis	Cardiology consult may review ECG and recommend verapamil, or scenario may end to allow additional time for discussion and debriefing

The scenario begins with the patient sitting on a hospital stretcher on a monitor, alert, and speaking, with no IV access. Her parent (a second facilitator if available) is present at the bedside to provide additional history. The patient's examination was notable for tachycardia and evidence of decreased perfusion as evident by diminished pulses, delayed capillary refill, and hypotension. Participants were expected to complete an evaluation including ECG and devise a differential diagnosis. The case concluded when they identified ECG findings concerning Belhassen tachycardia and administered the appropriate medical intervention, verapamil, or after 15 min elapsed since the onset of Belhassen tachycardia. If they proceeded with the management of SVT with adenosine or cardioversion, treatment was unsuccessful.

Debriefing

The guidelines available in Appendix A is used to facilitate debriefing sessions after the simulation. This tool allowed each facilitator to tailor the discussion based on the needs and performance of the participants. We began the debrief by allowing participants to provide general reflections on their experience followed by a discussion of the components of the case. Observations made by participants and facilitators were then used as lead points into discussions on teamwork, communication, as well as diagnostic and management skills. The didactic PowerPoint slides (Appendix B) were then briefly reviewed to provide additional information to reinforce the content of the scenario. Depending on the learner's experience level, the PowerPoint slides could also be presented before the scenario or reviewed by participants asynchronously in a flipped classroom model to prime participants for the scenario.

Assessment

Facilitators provided formative feedback to participants on their performance mapped to the learning objectives. All participants completed an evaluation form after the completion of the debriefing. Participants were asked to state their agreement with evaluative statements using a Likert scale (1=strongly disagree, 2=disagree, 3=neutral, 4=agree, 5=strongly agree). They were asked about their experience during the educational session and about their clinical confidence related to the learning objectives after participating in the session. They were also asked to answer free-response questions related to their experience.

Participants gave high ratings to the simulation (Table 2) and reported a high level of confidence with skills and knowledge related to the content after participating in the session (Table 3).

**Table 2:** Participants' experience during the simulation session (Likert scale: 1=strongly disagree, 3=neutral, 5= strongly agree); N=20

Participant survey question	Mean Likert score	Range
This simulation case provided is relevant to my work	4.7	4-5
The simulation case was realistic	4.6	3-5
This simulation case was effective in teaching basic resuscitation skills	4.7	3-5
The debrief created a safe environment	4.9	4-5
The debrief promoted reflection and team discussion	4.8	4-5

**Table 3:** Participants' clinical confidence after participating in the session (Likert scale: 1=strongly disagree, 3=neutral, 5=strongly agree); N=20 ECG, electrocardiogram; SVT, supraventricular tachycardia.

Participant survey question		Mean Likert score	Range
Perform a primary assessment of a pediatric patient with tachycardia	4.6		3-5
Correctly evaluate an ECG for findings that differentiate Belhassen tachycardia from SVT with aberrancy	4.2		3-5
Develop an appropriate management plan for a patient with Belhassen tachycardia	4.5		3-5
Evaluate the effectiveness of their interventions through patient reassessment including a repeat ECG	4.5		3-5
Demonstrate effective team leadership, team dynamics, and communication	4.5		3-5

Participants reported several ways in which the simulation session would change how they do their job and how the scenario could be improved. Their comments and implementation experience are summarized in Table 4.

**Table 4:** Participants' comments after participating in the scenario SVT, supraventricular tachycardia.

Implementation site	Participant comments
Site #1	Exposure to case increased depth of understanding of how to approach pediatric patients with tachycardia and medical decision-making when adenosine fails to abort SVT or suspected SVT
Site #2	Participants identified that the didactic PowerPoint reinforced knowledge learned during the simulation session. Participants suggested that it may be helpful to have didactic PowerPoint first to prime them for participation in the simulation scenario. This order could be considered depending on the experience level of participants
Site #3	Multiple participants commented that participation in the scenario would encourage them to consider a broader diagnosis for pediatric tachycardia in their clinical practice in the future. The scenario exposed them to a less common tachycardia that they were unfamiliar with from their clinical experience

## Discussion

The goal of the case was to challenge advanced learners who have experience treating patients using PALS algorithms, with the opportunity to manage a more nuanced case in a simulated environment while continuing to hone teamwork and communication skills. Belhassen tachycardia is unique in that it does not typically respond to the standard therapeutic measures for ventricular tachycardia, as outlined in PALS. Fortunately, characteristic findings on the ECG help differentiate it from other forms of wide complex tachycardia. This simulation allows participants to evaluate a simulated patient and trial therapeutic interventions in a safe learning environment.

Physicians caring for pediatric patients in emergency settings must be prepared to rapidly handle unexpected and rare presentations. They must be able to apply life-saving algorithms and be able to identify when illness patterns are falling outside of the expected course and respond appropriately. However, in practice, exposure to acutely ill patients and critical procedures within the pediatric emergency department is often limited [13,14]. To supplement traditional training, simulation can be used to teach and reinforce clinical and procedural skills [15]. Nearly all PEM fellowships within the United States incorporate simulation into their education [16]. The case described in this technical report can be incorporated into a longitudinal curriculum to challenge advanced learners to think beyond standard algorithms.

This simulation was implemented with learners from multiple institutions using the materials provided in this technical report. While the scenario was implemented with advanced trainees, it could also be run in an interdisciplinary setting with a combination of attending and trainee physicians, nurses, and respiratory therapists. If members of multiple disciplines are present, individuals should function in a role consistent with their role in a clinical setting. The proportion of time spent on the debrief and didactic slides as well as debrief topic emphasis may be adjusted to the learner's needs.

Participants rated their confidence related to the learning objectives high after participation. A limitation to the evaluation of this simulation is that we were unable to measure the actual clinical performance of learners after participation, given the extremely rare occurrence of rhythm disturbances in pediatric patients. Furthermore, we did not measure changes in knowledge after participation, as this is not routine practice during the standard fellow education. Participants expressed positive reactions to the session. Some learners provided feedback that the didactic slides could be provided before the case as a primer related to the content.

## Conclusions

Teaching advanced learners responsible for the emergency care of pediatric patients to identify arrhythmias that are unusual and do not respond to typical treatments outlined by PALS through simulation is a valuable experience. It allows learners to develop broad differentials, practice diagnostic reasoning, and trial interventions in an environment that is safe for patients and providers. Simulation as an instructional method also allows participants to engage in teamwork and practice communication skills that are crucial for patient care within the ED environment, regardless of the case. This technical report provides facilitators with the materials required to implement the simulation with learners at their institution.

## Figures and Tables

**Figure 4:**
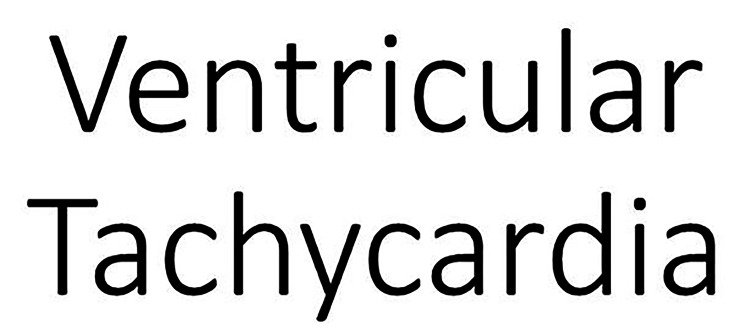
Slide 1

**Figure 5:**
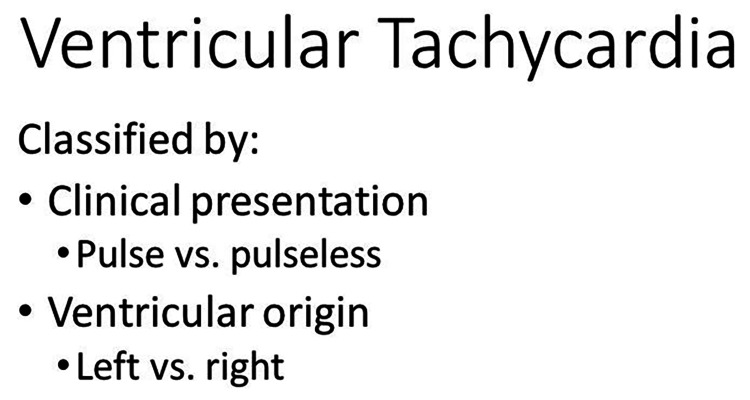
Slide 2

**Figure 6:**
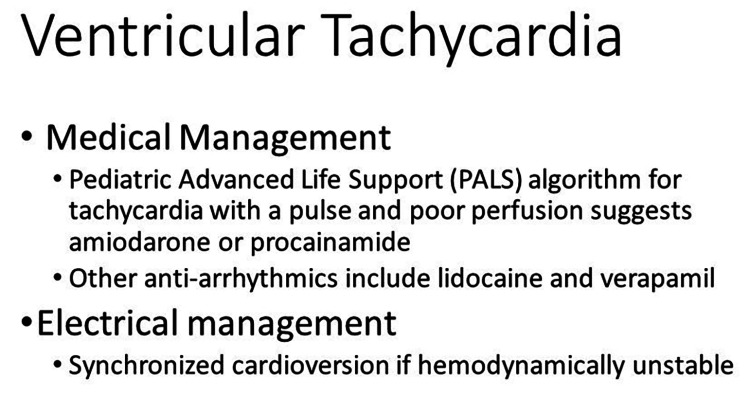
Slide 3 PALS, Pediatric Advanced Life Support.

**Figure 7:**
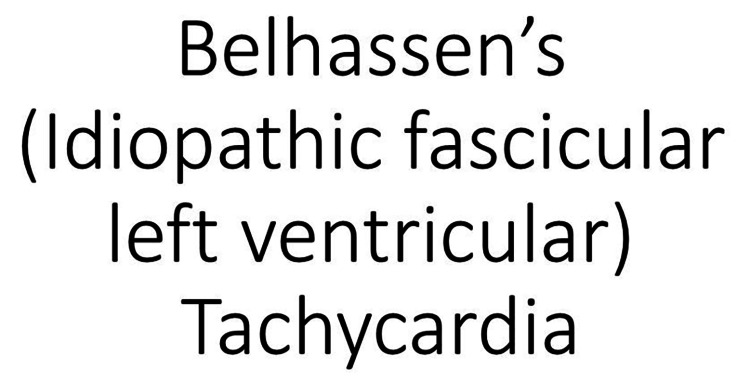
Slide 4

**Figure 8:**
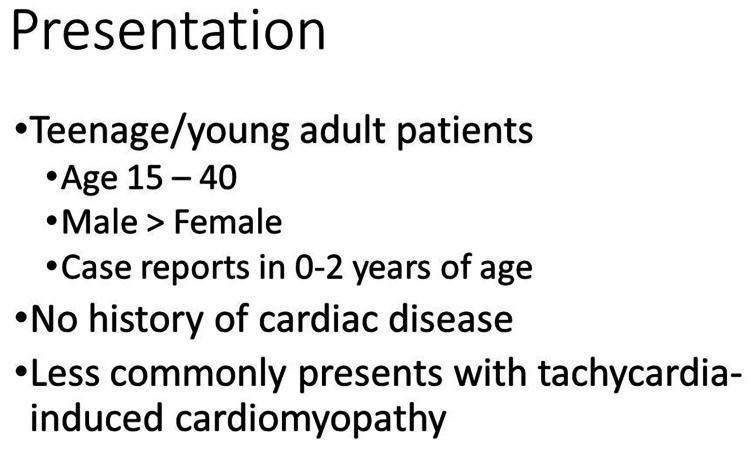
Slide 5

**Figure 9:**
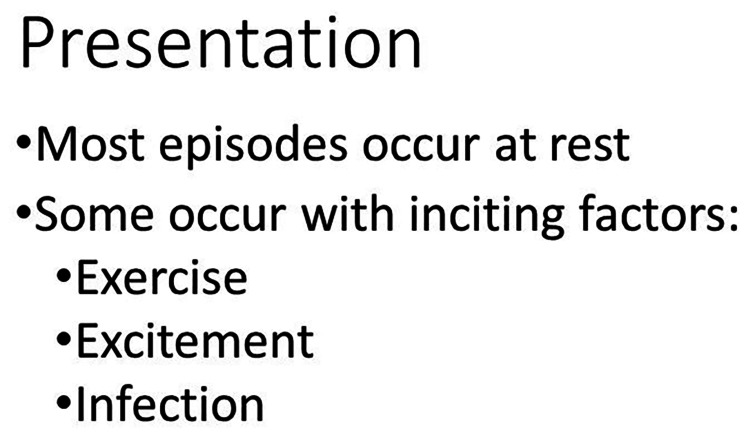
Slide 6

**Figure 10:**
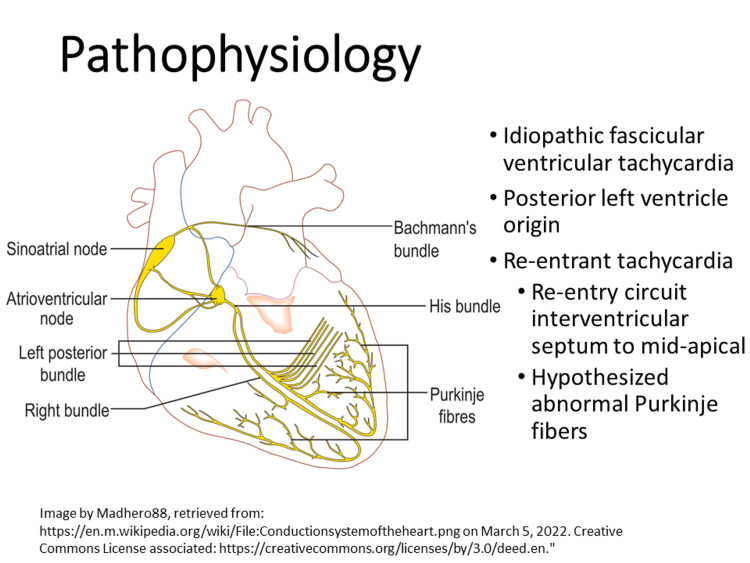
Slide 7 Permission to use this image is granted by the file's license under the Creative Commons Attribution 3.0 Unported license.

**Figure 11:**
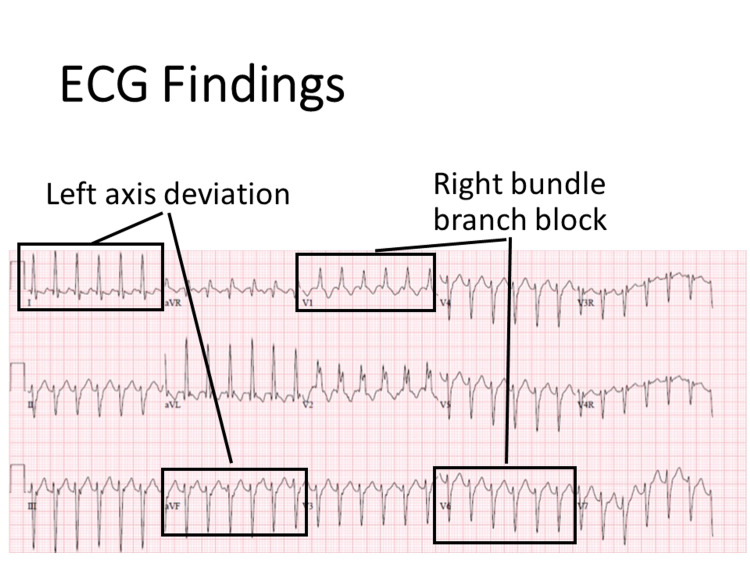
Slide 8 ECG, electrocardiogram.

**Figure 12:**
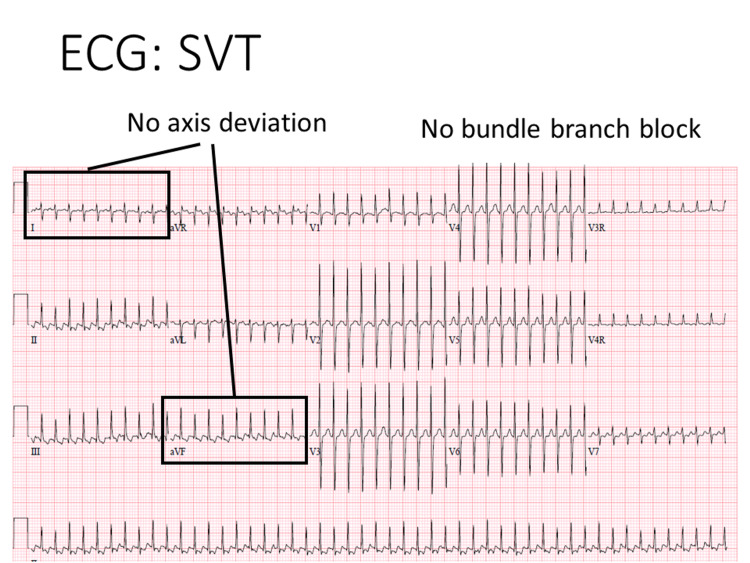
Slide 9 ECG, electrocardiogram; SVT, supraventriclar tachycardia.

**Figure 13:**
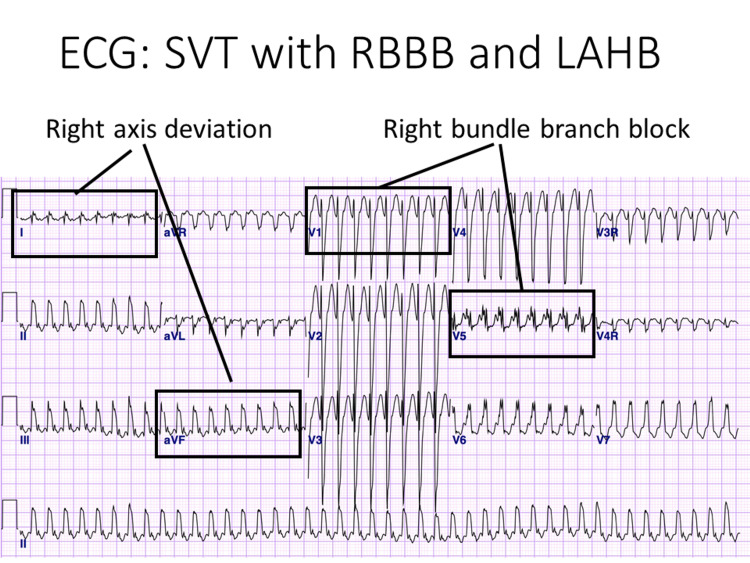
Slide 10 ECG, electrocardiogram; SVT, supraventricular tachycardia; RBBB, right bundle branch block; LAHB, left anterior hemiblock.

**Figure 14:**
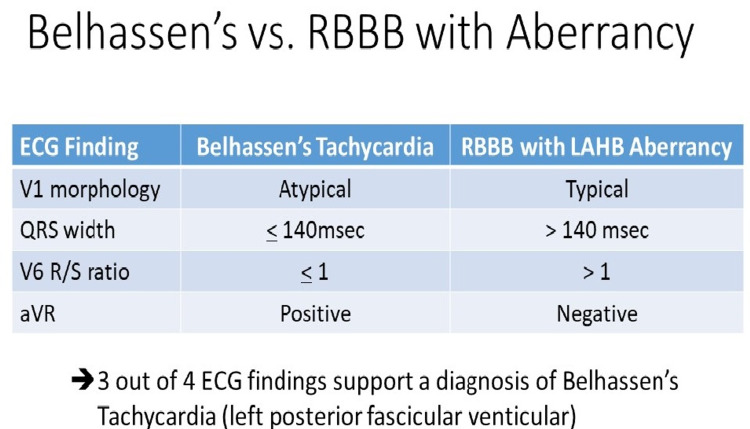
Slide 11 ECG, electrocardiogram; RBBB, right bundle branch block; LAHB, left anterior hemiblock. Source: [6].

**Figure 15:**
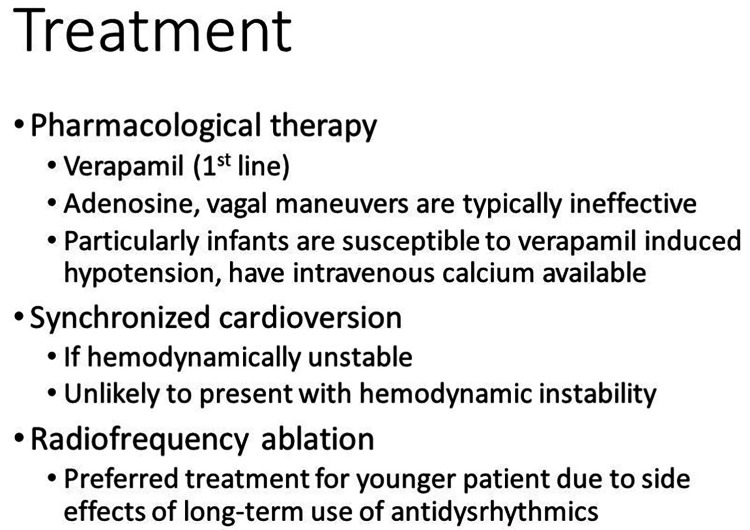
Slide 12

**Figure 16:**
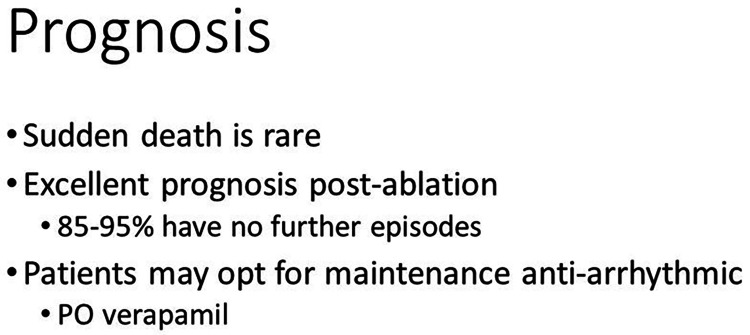
Slide 13

## Appendices

Appendix A: Belhassen Tachycardia Debriefing Guide

Debriefing overview

We believe that reflective learning occurs in the debrief. It is an opportunity for learners to reflect on their medical decision-making, technical, teamwork, and communication skills. The ultimate goal is to identify the gaps and potential solutions to close those gaps, leading to improved patient safety and better quality of care.

Framework for Debriefing:

We model our debriefing after PEARLS [1]. Each debrief typically has four phases. (1) Reactions phase: an opportunity for learners to express their emotional experience, where they may reveal key areas that are important to them. (2) Description phase: an opportunity for learners to summarize key events in the scenario to ensure that educators and learners are on the same page. (3) Analysis phase: opportunity to explore the medical decisions, technical, teamwork, and communication performance of the team. (4) Summary phase: a review of key take-home points, led by learners or educators.

General Debriefing Goals:

Following are the goals of a debriefing session: (i) creating a safe learning environment; (ii) normalizing gaps in performance, if at all possible; (iii) using open-ended questions rather than yes/no questions; and (iv) trying to facilitate the team’s discussion (avoid lecturing).

1)       Reactions phase

There are different perspectives on emotions and debriefing. One perspective is that learners may find it difficult to engage in the analysis of performance until emotions are addressed. A different perspective is that adult learners should process their emotions independently outside the context of the debrief.

Our perspective aligns with the first described. If a group or team member is feeling emotionally charged (e.g., ashamed, angry, or frustrated), it may be difficult to be actively engaged, receptive to feedback, and able to engage in learning, until the emotions are addressed.

What you might say to start the debrief:

-       “How did that feel?”

-       “How did that go for you?”

-       “What are your initial reactions?”

-       “How is everyone else feeling?”

2)       Description phase

Summary of key events to ensure that educators and participants are on the same page.

What you might say:

-       “Could someone summarize the case, so we are all on the same page?”

-       “From your perspective, what were the main issues you dealt with?"

3)       Analysis phase

Promote reflection on performance (medical decision-making, technical skills, teamwork, and communication) and identify opportunities for improvement.

What you might say:

-       “Let’s talk more about the case.”

-       “What aspects did your team manage well? Why?”

-       “What could your team manage better next time? Why?”

-       “I want to spend a couple of minutes talking about XXX. Can you tell me more about what was going on?”

-       "I noticed you [behavior]…next time you may want to [suggested behavior]… because [provide rationale]."

4)       Summary phase

Opportunity to review key learning points. Participants or educators can identify take-home points.

What you might say:

Medical management/technical skills examples:

-       “This was a scenario of a patient with ventricular tachycardia, Belhassen variant.”

-       “Signs and symptoms of Belhassen’s ventricular tachycardia include: chest pain, dizziness, and fatigue AND specific EKG findings: left axis deviation and right bundle branch block.”

-       “Formulating a list of possible diagnoses is critical to identifying an etiology and determining a treatment plan.”

-       “Evaluation of ventricular tachycardia includes: evaluating the ABCDEs and obtaining an ECG.”

-       “Management of Belhassen’s ventricular tachycardia includes: treatment of ABCDEs and verapamil.”

Teamwork/communication examples:

-       Recognize the need for a full resuscitation team when a patient has a potentially unstable arrhythmia.

-       Designate team member roles including the leader to support coordination and team functioning.

-       Role assignment to specific individuals to avoid duplication/omission of tasks.

-       Respect all team members is key to enable empowerment to speak up if patient safety issues arise.

-       Use briefs and/or huddles to create a shared mental model for the working diagnosis and management plan.

-       Closed-loop communication is of paramount importance to ensure safe and adequate communication.

Debriefing guide

Below are examples of learning objective-based statements and questions you may use to debrief the team.

**Table 5:** Debriefing guide ABCDE, airway, breathing, circulation, disability, exposure; ECG, electrocardiogram.

Examples of debriefing for different learning objectives		
Assess a patient with tachycardia		
Debriefer script	Reference material	Instructor notes
I noticed you (were complete/missed some opportunities) in performing your initial evaluation - ABCDEs. This was (great/could have been even better) because early identification and management could lead to improved outcomes. How did your team decide on the evaluation priorities? What helped/hindered you? I saw you (were quick/took a while) to identify ventricular tachycardia in your differential diagnosis. This (was great/could have been even better) since delays in recognition can result in clinical deterioration. What were you considering in your differential diagnosis? What helped/hindered you from considering other options?	Components of an initial evaluation: primary survey (ABCDEs), vital signs; secondary survey; differential diagnosis for tachycardia with poor perfusion: arrhythmia, sepsis, hypovolemia	
Identify ventricular tachycardia		
Debriefer script	Reference material	Instructor notes
I noticed you (were quick/took a while) to identify Belhassen’s ventricular tachycardia as the rhythm. This was (great/could lead to delays) since delays in recognition can result in clinical deterioration. What were your thoughts/priorities? What helped/hindered you from identifying Belhassen’s ventricular tachycardia? How did you distinguish Belhassen’s ventricular tachycardia from other tachycardias?	Initial management of ventricular arrhythmia with poor perfusion: obtain ECG, identify the rhythm, treat with appropriate anti-arrhythmic, anticipate decompensation. Differentiating Belhassen’s: rSR’ V1 morphology, QRS width, positive QRS in aVR and the V6 R/S, left axis deviation, does not respond to adenosine	
Identify ventricular tachycardia		
Debriefer script	Reference material	Instructor notes
I noticed you (were quick/took a while) to treat Belhassen’s ventricular tachycardia. This was (great/could lead to delays) since delays in management can result in clinical deterioration. What were your thoughts/priorities? How did you determine which medications to give?		
Reassess after intervention		
Debriefer script	Reference material	Instructor notes
I noticed you (were quick/could have been quicker) to obtain a repeat ECG and repeat set of vital signs when the rhythm changed. This (was great/could have been better) because reassessing the patient is key in determining the next steps in management. What were your thoughts/priorities after you stabilized the patient? What helped/hindered you?	Evaluation of the patient after change in the rhythm: vital sign changes, examination changes, ECG changes	

Examples for debriefing teamwork learning objectives			
Roles and responsibilities			
Debriefer script	Reference material		Instructor notes
Let us talk about how you functioned as a team. From my perspective, it looked like you (did/did not) have a clear team leader and defined team roles. I think this is (great/concerning) because clear team roles can help a team function smoothly - improving how quickly interventions take place and reducing errors. How did you function as a team? What did you think about your roles?	Team leader: clear direction, coordination, timely interventions foot of the patient. Airway MD: manage the airway at head of the patient. Survey MD: primary survey, secondary survey, pulses, reassessments. Nursing roles: medication prep (draw-up meds), medication admin (give meds), documenting (timekeeper)		
Brief and huddle			
Debriefer script		Reference material	Instructor notes
I noticed that your team (did/did not/took a while to) (brief before the initial patient assessment/huddle after the initial evaluation). I thought this was (great/could have helped you work better as a team) to facilitate patient care. What (helped/hindered) your team from (briefing/huddling)? How did that impact your team? What could your team have done differently? How can you make sure that (does/does not) happen again?		The goal of a brief/huddle is to create a shared mental model. Assure all team members know what the working diagnosis is, management priorities, and next steps in care. Everyone on the team is responsible for making this happen. Anyone can ask for a brief/huddle. Brief/huddle is usually led by the team leader. If one team member does not know what is up or what is next, s/he is probably not alone.	

Directed call out			
Debriefer script	Reference material		Instructor notes
I noticed that you (did/did not/intermittently) used (people's names/roles/eye contact) when (calling out orders/asking for assistance). I thought this was (great/could have been more directed) to facilitate communication. What did you notice about orders/questions that were asked? How did this impact your team?	Directed call out. Tactical communication skills to assure that important orders/questions are specifically directed to one individual (rather than called out into the air). Example: “Jonathan - What is the SaO2%?” “Kim - Give normal saline 500 mL” “Team leader - she stopped responding to pain”		
Closed-loop communication/check back			
Debriefer script		Reference material	Instructor notes
I noticed that you used closed-loop communication (consistently/a lot/rarely). Closed-loop communication can be critical for catching errors and assuring that (information/an order/a request) is heard. How were the communication loops in the team? How did that impact your team? Has anyone seen problems with this in a patient resuscitation? Has anyone seen closed-loop communication prevent an error? How could you do it differently next time?		Closed-loop communication/check back is a strategy that requires verification of information. This enables the sender of the message to verify that it has been heard and heard correctly. It enables the receiver to confirm what they heard is correct. Team leader, “Call for ECG.” Float nurse, “calling technician for an ECG.” Team leader, “correct”	

Appendix B: Belhassen Tachycardia Didactic Slides
